# DNA double-strand breaks in the *Toxoplasma gondii*-infected cells by the action of reactive oxygen species

**DOI:** 10.1186/s13071-020-04324-7

**Published:** 2020-09-25

**Authors:** Haohan Zhuang, Chaoqun Yao, Xianfeng Zhao, Xueqiu Chen, Yimin Yang, Siyang Huang, Lingtao Pan, Aifang Du, Yi Yang

**Affiliations:** 1 Institute of Preventive Veterinary Medicine, Zhejiang Provincial Key Laboratory of Preventive Veterinary Medicine, College of Animal Sciences, Zhejiang University, Hangzhou, 310058 PR China; 2 Departments of Biomedical Sciences and One Health Center for Zoonoses and Tropical Veterinary Medicine, Ross University School of Veterinary Medicine, P.O. Box 334, Basseterre, West Indies Saint Kitts and Nevis; 3 Animals & Plant Inspection and Quarantine Technology Center of Shenzhen Customs, Shenzhen, 518045 PR China; 4 Institute of Comparative Medicine, College of Veterinary Medicine, Yangzhou University, and Jiangsu Co-innovation Center for Prevention and Control of Important Animal Infectious Diseases and Zoonosis, and Jiangsu Key Laboratory of Zoonosis, Yangzhou, 225009 Jiangsu Province PR China

**Keywords:** *Toxoplasma gondii*, DNA damage, Reactive oxygen species, DNA damage response

## Abstract

**Background:**

*Toxoplasma gondii* is an obligate parasite of all warm-blooded animals around the globe. Once infecting a cell, it manipulates the host’s DNA damage response that is yet to be elucidated. The objectives of the present study were three-fold: (i) to assess DNA damages in *T. gondii*-infected cells *in vitro*; (ii) to ascertain causes of DNA damage in *T. gondii*-infected cells; and (iii) to investigate activation of DNA damage responses during *T. gondii* infection.

**Methods:**

HeLa, Vero and HEK293 cells were infected with *T. gondii* at a multiplicity of infection (MOI) of 10:1. Infected cells were analyzed for a biomarker of DNA double-strand breaks (DSBs) γH2AX at 10 h, 20 h or 30 h post-infection using both western blot and immunofluorescence assay. Reactive oxygen species (ROS) levels were measured using 2′,7′-dichlorodihydrofluorescein diacetate (H2DCFDA), and ROS-induced DNA damage was inhibited by a ROS inhibitor N-acetylcysteine (NAC). Lastly, DNA damage responses were evaluated by detecting the active form of ataxia telangiectasia mutated/checkpoint kinase 2 (ATM/CHK2) by western blot.

**Results:**

γH2AX levels in the infected HeLa cells were significantly increased over time during *T. gondii* infection compared to uninfected cells. NAC treatment greatly reduced ROS and concomitantly diminished γH2AX in host cells. The phosphorylated ATM/CHK2 were elevated in *T. gondii*-infected cells.

**Conclusions:**

*Toxoplasma gondii* infection triggered DNA DSBs with ROS as a major player in host cells *in vitro*. It also activated DNA damage response pathway ATM/CHK2. *Toxoplasma gondii* manages to keep a balance between survival and apoptosis of its host cells for the benefit of its own survival. 
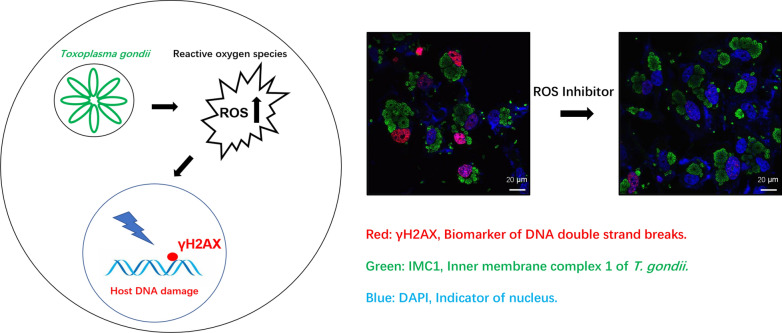

## Background

The protozoan parasite *Toxoplasma gondii* infects almost all warm-blooded animals including humans worldwide [1]. It modulates some biological processes of the infected cell, such as autophagy and apoptosis, to facilitate its survival and proliferation [2, 3]. DNA damage was also reported in the retina and the peripheral blood cells of *T. gondii*-infected mice [4, 5]. However, it remains to be elucidated how *T. gondii* causes host DNA damage and what the DNA damage responses are. DNA damage appears in different forms such as single-strand breaks (SSBs), double-strand breaks (DSBs), missing bases and chemical modification of bases, etc. [6]. DNA damage may be caused by various endogenous or exogenous factors. Examples are reactive oxygen species (ROS) and ultraviolet light and chemical reagents, respectively [7].

ROS are naturally generated from metabolic and biochemical reactions and are a major source of the endogenous stress [8]. Superoxide, hydrogen peroxide, hydroxyl radicals and singlet oxygen are all ROS. High levels of ROS can oxidize DNA molecules leading to base oxidization, SSBs and DSBs [9]. Increasing lines of evidence clearly show that ROS are triggered by bacterial, viral and parasitic pathogens in host cells during infection [10]. Further, ROS affect DNA integrity of host cells although it is debatable whether ROS are always detrimental to infecting pathogens [11].

According to its virulence in the mouse model, the protozoan *T. gondii* is divided into three vastly different virulent groups, i.e. Type I, II and III. Interestingly, infections by both avirulent Type III (CTG strain) and virulent Type I (GT1 strain) trigger ROS production in the infected macrophages. CTG-strain parasites are mainly cleared by the naive macrophages in a manner depending on NADPH oxidase-generated ROS without activation of interferon gamma [12]. Due to ROS’ potential detrimental impact on DNA, we explored the association between elevated ROS levels and host DNA damage during *T. gondii* infection. We used γH2AX, a well-characterized marker for DNA DSBs, to demonstrate DNA damage in *T. gondii*-infected HeLa cells. γH2AX levels increased over time during *T. gondii* infection and ROS was the major player in the DNA DSBs. Furthermore, the DNA damage response pathway ataxia telangiectasia mutated/checkpoint kinase 2 (ATM/CHK2) was activated, suggesting roles of DNA damage in regulation of other biological processes as well in these *T. gondii*-infected host cells. Together, our data reveal the major role of ROS in host DNA damage during *T. gondii* infection, and lay a foundation for better understanding how *T. gondii* interacts with its host from the view of DNA damage.

## Methods

### Cells, parasites and mice

Vero, HEK293T and HeLa cells were all obtained from the Cell Bank of the Chinese Academy of Sciences (Shanghai, China). The cells were cultured in DMEM (Biological Industries, Kibbutz, Israel) supplemented with a final concentration of 10% fetal bovine serum (FBS) (Biological Industries) and 1% penicillin-streptomycin-glutamine (Gibco, Carlsbad, USA). All cells were treated with MycAway^TM^ elimination reagent (Yeason, Shanghai, China), followed by testing using One-Step Mycoplasma Detection Kit (Yeason) to make sure they were *Mycoplasma* free. Both the *T*. *gondii* RH*Δku80* strain and EGFP-RH*Δku80* strain were serially passaged as tachyzoites in Vero cells in our own laboratory as previously described [13]. Briefly, tachyzoites were collected in the supernatant of centrifugation of spontaneously ruptured *T. gondii*-infected Vero cells at 300*g* for 5 min at room temperature. They were further cleaned by passing through a syringe filter of 5-μm pore size (Millipore, Darmstadt, Germany). Parasites were used to infect fresh Vero cells at a multiplicity of infection (MOI) of 10:1 after quantification using a hemocytometer. Six-week-old BALB/c mice were obtained from Shanghai SLAC Laboratory Animal Co., Ltd (Shanghai, China), and housed in a ventilated cage kept under a conditioned temperature of 25 °C with a light/dark cycle of 14 h/10 h.

### Antibodies and mouse sera

The antibodies to the following molecules were purchased from various sources as indicated. γH2AX (20E3, Cell Signaling Technology, Danvers, USA) was used to detect DNA DSBs. Actin and the cleaved caspase 3 (Abcam, Cambridge, UK) were used as a loading control and apoptotic marker, respectively. ATM, ATM-S1981, CHK2 and CHK2-T68 (Cell Signaling) were used to document DNA damage responses. HRP-conjugated goat anti-mouse IgG and anti-rabbit IgG (Fude, Hangzhou, China) were used in the western blot. Donkey anti-mouse Alexa Fluor 488 and goat anti-rabbit Alexa Fluor 594 (Invitrogen, Carlsbad, USA) were used in the immunofluorescence assay (IFA). Anti-*T. gondii* inner membrane complex 1 (IMC1) and surface protein 1 (SAG1) mouse sera, kind gifts from colleagues Miss Mi Lin and Miss Mingxiu Zhao (College of Animal Sciences, Zhejiang University), were used to detect *T. gondii* in IFA and western blot, respectively. *Toxoplasma gondii* positive serum was harvested from BALB/c mice 6 days post-infection (pi) and used to block *T. gondii* invasion into HeLa cells. Negative serum was collected from the same mice prior to infection and used as controls.

### Western blot

Western blot was carried out as previously described [13]. Briefly, cells were lysed by incubation for 30 min in ice-cold radioimmunoprecipitation assay lysis buffer supplemented with a protease inhibitor cocktail (Bimake, Shanghai, China). The soluble proteins derived from the supernatants of the centrifugation of the cellular lysates at 12,000*g*, 4 °C for 10 min were measured using a BCA assay kit (Fude, Hangzhou, China) and were then subjected to SDS-PAGE. The 0.22 μm PVDF membrane (Millipore, Darmstadt, Germany) blotted with proteins was blocked with 5% skimmed milk (Sangon, Shanghai, China) in Tris buffered saline containing 0.5% Tween-20 (TBST) followed by incubation in suitable primary antibodies. The membranes were then probed with appropriate HRP-conjugated secondary antibodies (Fude). Membranes were rinsed thrice in TBS (10 min each) at each interval. Signals were immediately documented using the ChemiDoc™ chemiluminescence system (Bio-Rad, Hercules, USA) after membranes were exposed to ECL substrates (Fude).

### IFA

IFA was carried out as previously described [12]. Briefly, cells grown on coverslips in a 24-well plate were fixed with 4% paraformaldehyde in phosphate-buffered saline (PBS) for 10 min and permeabilized with 0.25% triton-PBS for 10 min. They were incubated with the primary antibodies for 1 h after being blocked in 1% bovine serum albumin for 1 h followed by submerging in suitable Alexa-fluor-conjugated secondary antibodies for 1 h in the dark. Afterwards they were counterstained with DAPI (Sigma-Aldrich, Saint Louis, USA) for 1 min. At each interval, the cells were rinsed thrice with PBS (10 min each). The coverslips were then mounted to a glass slide and cell images were obtained using an Olympus IX81 FV1000 confocal microscope (Olympus, Monolith, Japan).

### ROS measurement

ROS measurement and inhibition was carried out as previously described [14, 15]. Briefly, HeLa cells were seeded on 24-well plates and infected with *T. gondii* RH*Δku80* strain parasites at a multiplicity of infection (MOI) of 10:1. Twenty-four hours post-infection (hpi), infected cells were treated with the ROS inhibitor N-acetylcysteine (NAC) at 50 μm for 1 h, 2 h or 4 h. NAC, a precursor of cysteine and glutathione, cellularly functions as an antioxidant *via* the redox potential of thiols or *via* increasing glutathione levels [16]. Following NAC treatment and after being rinsed 3 times in PBS, infected cells were incubated in 10 μm 2′,7′-dichlorodihydrofluorescein diacetate (H2DCFDA; MCE, Monmouth, USA) in the dark at 37 °C and 5% CO_2_ for 30 min. H2DCFDA is a non-fluorescent precursor that is intracellularly oxidized by ROS to form a highly fluorescent product DCF. Finally, fluorescence signals of cells were measured using a Synergy 2 plate reader (Biotek, Winooski, USA) at 485 nm exiting wavelength.

### Quantification and statistical analysis

Quantification of the western-blot protein bands was performed by Image J. Western blot data from Image J and fluorescence data from a plate reader representing triplicate samples were analyzed by GraphPad Prism 7.0 (GraphPad, La Jolla, USA). Statistical analysis was performed by one-way ANOVA and Student’s t-test with *P* ≤ 0.05 being considered significant.

## Results

### *Toxoplasma gondii* infection triggered DNA DSBs in host cells *in vitro*

**Fig. 1:**
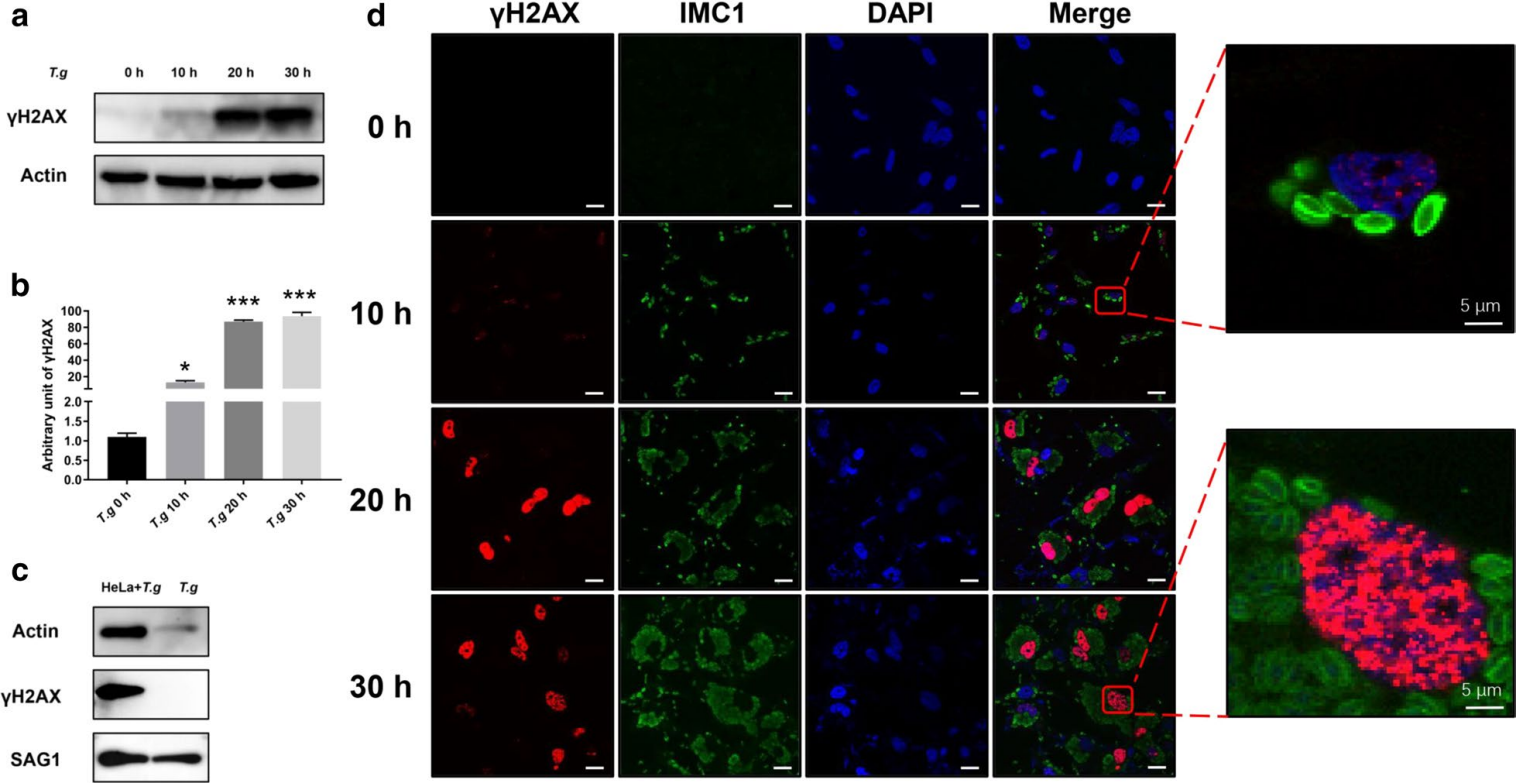
DNA damage of the *T. gondii*-infected host cells *in vitro*. Antibodies to γH2AX and actin, SAG1 serum and IMC serum were all used at a 1:1000 dilution for the western blot and at a 1:200 dilution for the immunofluorescence assay (IFA). **a** HeLa cells were infected with *T. gondii* RH*Δku80* at a MOI of 10:1. γH2AX levels were detected by western blot at 0 h (uninfected control), 10 h, 20 h or 30 h post-infection. Actin was used as a loading control. **b** Quantification of γH2AX shown in **a**. Mean  ±  SD (*n* = 3) of γH2AX levels are shown with that of uninfected cells set as one arbitrary unit. **c** γH2AX levels were measured by western blot in *T. gondii*-infected HeLa cells and purified *T. gondii* parasites that were freshly isolated from infected Hela cells. Actin and SAG1 were used as a loading control for host cells and *T. gondii*, respectively. **d** γH2AX of uninfected (0 h) and infected HeLa cells at 10 h, 20 h or 30 h post-infection by IFA. IMC1 mouse serum and DAPI were used to indicate *T. gondii* and nuclei, respectively. *Key*: red, γH2AX; green, IMC1; blue, DAPI. All experiments were performed in triplicate. *P ≤ 0.05, ***P ≤ 0.001. *Scale-bars*: 30 μm

DSBs are one of the most common forms of DNA damage [17]. Phosphorylation at the ser139 position of H2AX named γH2AX is a well-characterized DSBs marker of the mammalian cells [18]. To study host cell DNA damage by *T. gondii* infection *in vitro*, we infected Vero, HEK293T or HeLa cells with *T. gondii* RH*Δku80* strain parasites at a MOI of 10:1. A similar effect was observed among all the three types of cells upon *T. gondii* infections. Hela cells were used throughout the entire study description here unless another cell type was specifically identified. The cells were harvested at 0 h (uninfected control) 10, 20 or 30 hpi and analyzed by western blot to detect γH2AX. Compared to 0 h, γH2AX levels in the infected HeLa cells at 10 h, 20 h, and 30 h had increased approximately 10-, 85- and 90-fold (10 h: ANOVA, *F*_(3, 8)_ = 13.87, *P* = 0.0323; 20 h: ANOVA, *F*_(3, 8)_ = 13.87, *P* = 0.0002; 30 h: ANOVA, *F*_(3, 8)_ = 13.87, *P* = 0.0005) (Fig. 1a, b), respectively. A similar trend was observed in the infected Vero and HEK293T cells as well (data not shown). We next determined the source of γH2AX since Fig. 1a, b did not show whether it originated from the infected host cells, parasites themselves or both. Tachyzoites were harvested by passing the infected Hela cells at 24 hpi through a 27-gauge syringe needle numerous times. They were then cleaned up with a 5-μm filter followed by cellular lysis by sonication. Their γH2AX levels were contrasted to those of the infected cells by western blot. γH2AX was abundantly detected in the infected HeLa cells. In contrast, γH2AX levels in parasites themselves were below the detectable level (Fig. 1c). These data clearly show that γH2AX predominately originated from the host cells rather than the parasites. To confirm γH2AX location in *T. gondii* infected cells, we carried out IFA using anti-IMC1 mouse serum and antibody to γH2AX to pinpoint *T. gondii* and DNA damage, respectively. Relative to uninfected cells, highly elevated γH2AX signals were detected in the nuclei of *T. gondii-*infected HeLa cells 20 or 30 hpi (Fig. 1d). Taken together, our data unequivocally demonstrated that *T. gondii* infection induced DSBs in host cells *in vitro.*

### *Toxoplasma gondii* induced DSBs were irrelevant to apoptosis and depended on its invasion

**Fig. 2:**
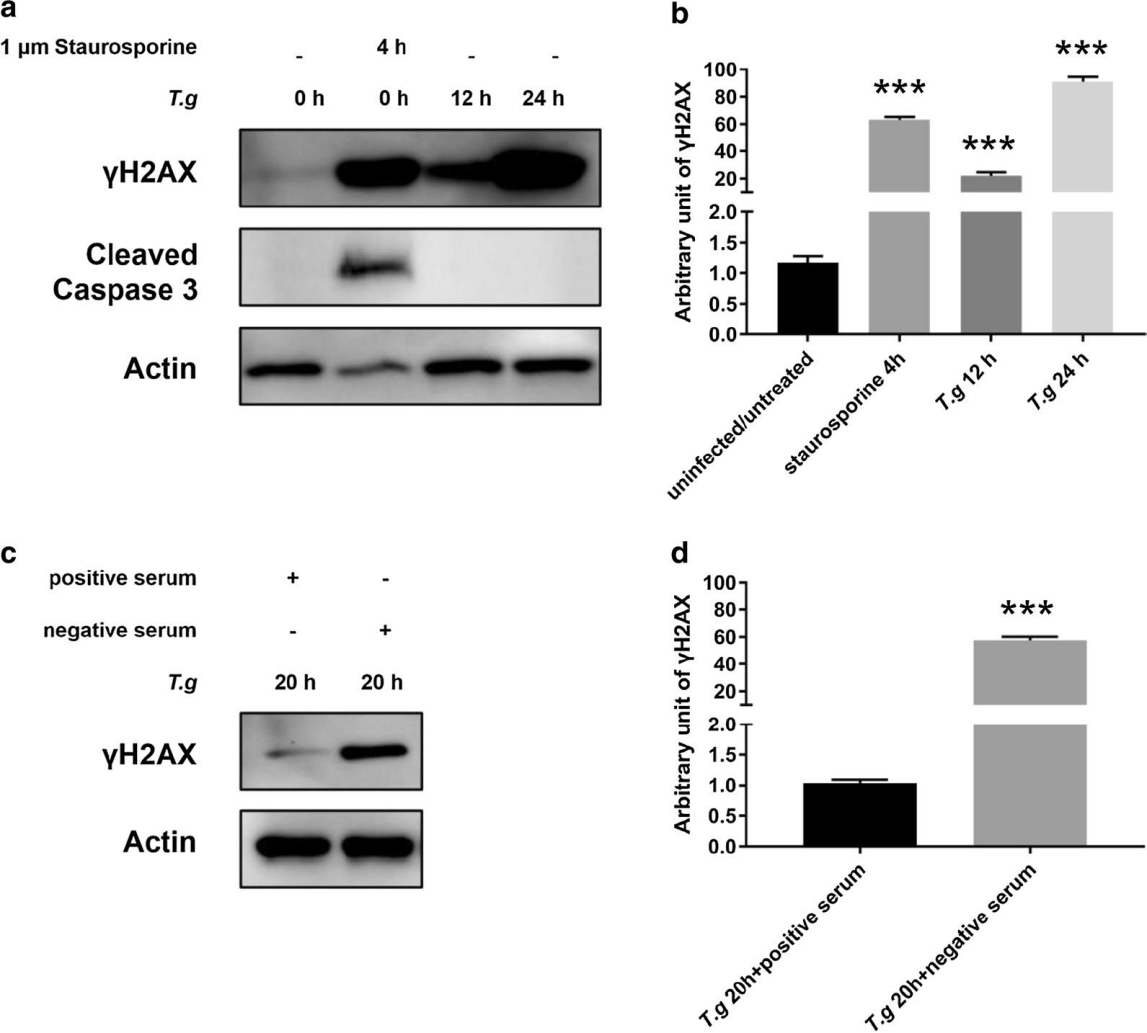
DNA damage of *T. gondii*-host cells upon its invasion. Antibodies to γH2AX, actin, and the cleaved caspase 3 were all used at a 1:1000 dilution. **a** Uninfected HeLa cells were treated with 1 μm staurosporine for 4 h to induce apoptosis. Treated, uninfected and infected HeLa cells were harvested 12 h or 24 h later, γH2AX and the cleaved caspase 3 were detected by western blot. **b** Quantification of γH2AX shown in **a**. Mean ± SD (*n* = 3) of γH2AX levels are shown with that of uninfected cells set as one arbitrary unit. **c** γH2AX was detected by western blot on HeLa cells 20 h post-infection with positive or negative serum-pretreated EGFP-RH*Δku80*. **d** Quantification of γH2AX shown in **c**. Mean ± SD (*n* = 3) of γH2AX levels are shown with that of uninfected cells set as one arbitrary unit. All experiments were performed in triplicate. ***P ≤ 0.001

It has been reported that γH2AX occurs in early apoptosis when DNA fragmentation just starts [19]. *Toxoplasma gondii* initiates apoptosis in some type of cells whereas it inhibits the process in others [20]. Our next experiments tested whether apoptosis influenced γH2AX levels during *T*. *gondii* infection. To this end, HeLa cells were treated with staurosporine, an apoptosis inducer [21], at 1 μm for 4 h and apoptosis was monitored by an early apoptotic signal, the cleaved caspase 3 in the western blot [22]. While γH2AX were detected in both staurosporine-treated HeLa cells and *T. gondii*-infected (12 h and 24 h) HeLa cells, the cleaved caspase 3 was only detected in staurosporine-treated cells with no trace of it being detected in *T. gondii-*infected cells (treated: ANOVA: *F*_(3, 8)_ = 80.51, *P* = 0.0003; 12 h: ANOVA: *F*_(3, 8)_ = 80.51, *P* = 0.0002; 24 h: ANOVA: *F*_(3, 8)_ = 80.51, *P* = 0.0003) (Fig. 2a, b). These data clearly showed that *T*. *gondii* infection did not trigger apoptosis in HeLa cells, which unequivocally ruled out that apoptosis was involved in their DNA damage.

During early infection, *T. gondii* invades host cells and leads to the formation of a parasitophorous vacuole to facilitate its proliferation [23]. We next examined whether *T. gondii* triggered host DNA damage before invasion. To this end, EGFP-RH*Δku80 T. gondii* tachyzoites were incubated with either positive serum from *T. gondii*-infected BALB/c mice or negative control serum for 1 h at 37 °C prior to infection as previously reported [24]. The positive serum was used to block the invasion of *T. gondii*. HeLa cells were then infected with serum-treated *T. gondii* at a MOI of 10:1, infected cells were imaged by fluorescence microscopy to detect EGFP 24 hpi or harvested for western blot analysis of γH2AX at 20 hpi. Compared with negative serum treatment, positive serum treatment dramatically blocked invasion of *T. gondii*, and decreased γH2AX levels by approximately 98%. (Student’s t-test: *t*_(2)_ = 17.91, *P* = 0.0002) (Fig. 2c, d). These results strongly indicate that induction of host DNA damage depends on the invasion of *T. gondii*.

### ROS contributed to host DNA damage

**Fig. 3:**
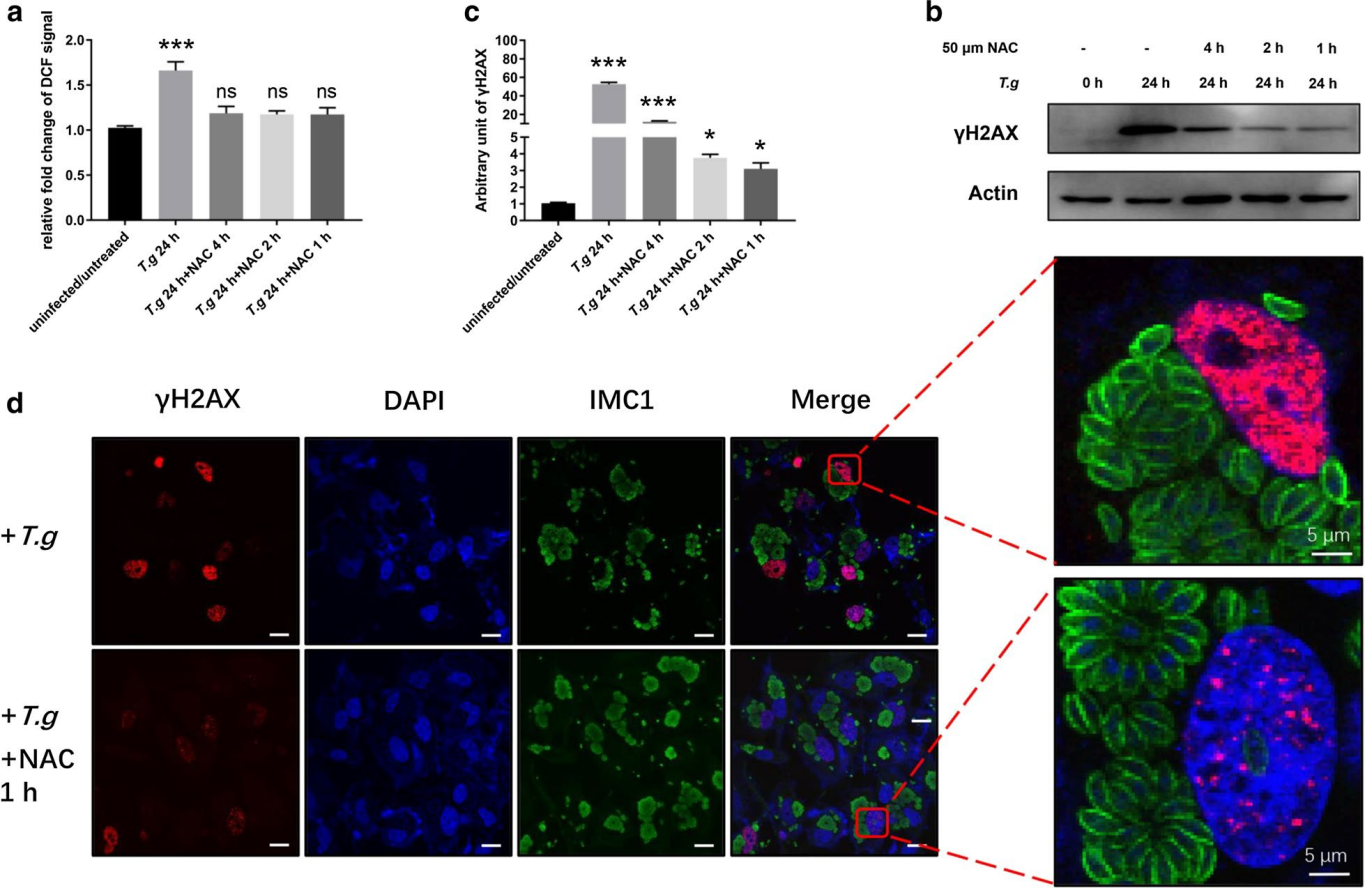
Reactive oxygen species contributed to DNA damage. HeLa cells were seeded in a 24-well plate and infected with *T. gondii* RH*Δku80* at a MOI of 10:1 for 24 h. N-acetylcysteine (NAC) at 50 μm was then applied to the infected cells for 1, 2 or 4 h. Antibodies to γH2AX, actin, and IMC serum were all used at a 1:1000 dilution for the western blot and at a 1:200 dilution for the immunofluorescence assay (IFA). **a** NAC treated *T. gondii*-infected cells were stained with 10 μm H2DCFDA and fluorescence signals were recorded. Data are presented as the mean ± SD (*n *= 3). **b** γH2AX was detected by western blot. **c** Quantification of γH2AX shown in **b**. Mean ± SD (*n *= 3) of γH2AX levels are shown with that of uninfected cells set as one arbitrary unit. **d** γH2AX assayed by IFA. Key: red, γH2AX; green, IMC1; blue, DAPI. All experiments were performed in triplicate. *P ≤ 0.05, ***P ≤ 0.001; ns: not significant. *Scale-bars*: 30 μm

ROS induce oxidative stress, which turns out to cause damages to macromolecules such as DNA, proteins and lipids [25]. Multiple studies have suggested pathogen-induced ROS role in host-cell DNA damage [26–30]. Elevated ROS levels have been reported in *T*. *gondii-*infected host cells [12, 31]. We hypothesized that ROS cause host DNA damage during *T. gondii* infection. To test this hypothesis, we treated *T*. *gondii* infected HeLa cells with N-acetylcysteine (NAC), a ROS inhibitor. NAC was applied to the infected cells 24 hpi for up to 4 h. Two methods were used to gauge the impact of ROS on host DNA damage. First, ROS levels of uninfected cells, host cells and NAC-treated host cells were analyzed using H2DCHFA, a non-fluorescent precursor that is converted to fluorescent DCF by ROS. H2DCHFC has been widely used as a probe to detect ROS for 50 years. It detects hydrogen peroxide, hydroxyl radicals and singlet oxygen but not superoxide anion [32]. The higher the ROS level is, the stronger florescence signal it produces. The second method was to monitor generation of γH2AX by western blot and IFA. The results show that ROS levels in the infected Hela cells increased by approximately 70% compared to those of uninfected cells 24 hpi by fluorescence quantification. NAC treatment for as little as 1 h completely offset the influence of *T. gondii* infection by bringing the ROS levels down almost to the basic level of the uninfected cells (infected: ANOVA: *F*_(4, 10)_ = 38.85, *P* = 0.0002; NAC 4 h: ANOVA: *F*_(4, 10)_ = 38.85, *P* = 0.8755; NAC 2 h: ANOVA: *F*_(4, 10)_ = 38.85, *P* = 0.9321; NAC 1 h: ANOVA: *F*_(4, 10)_ = 38.85, *P* = 0.8652) (Fig. 3a). Western blot showed that γH2AX levels in the NAC-treated infected host cells reduced by approximately 85%, 85% and 70% at 1, 2 and 4 h of NAC treatment, respectively, compared to those of the untreated infected host cells (infected: ANOVA: *F*_(4, 10)_ = 47.55, *P* = 0.0002; NAC 4 h: ANOVA: *F*_(4, 10)_ = 47.55, *P* = 0.0005; NAC 2 h: ANOVA: *F*_(4, 10)_ = 47.55, *P* = 0.0341; NAC 1 h: ANOVA: *F*_(4, 10)_ = 47.55, *P* = 0.0325) (Fig. 3b, c); quantification of protein bands were performed by Image J. IFA confirmed this dramatic reduction in γH2AX levels in NAC-treated host cells compared to untreated host cells. Further, γH2AX, although weak, remained detectable in these NAC-treated cells (Fig. 3d). These two lines of evidence suggested that diminishing ROS by NAC greatly reduced γH2AX levels in infected cells, indicating that ROS leads to DNA damage during *T. gondii* infection.

### Host DNA damage response pathway ATM/CHK2 was activated by *T. gondii* infection

**Fig. 4:**
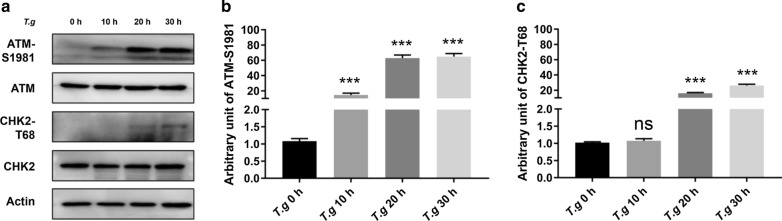
ATM/CHK2 activation upon *T. gondii* infection. **a** HeLa cells were infected with *T. gondii* RH*Δku80* at a MOI of 10:1. Expression of ATM, ATM-S1981, CHK2 and CHK2-T68 were detected by western blot at 0 h (uninfected control), 10 h, 20 h or 30 h post-infection. All antibodies were used at a 1:1000 dilution. **b** Quantitation of ATM-S1981 shown in **a** with ATM-S1981 levels of uninfected cells set as one arbitrary unit. **c** Quantitative data of CHK2-T68 shown in **a** with CHK2-T68 levels of uninfected cells set as one arbitrary unit. Data are presented as the mean ± SD (*n* = 3). All experiments were performed in triplicate. ***P ≤ 0.001; ns: not significant

DNA damage response is triggered upon DNA damage in the eukaryotic cells. A network of kinase pathways is involved in this biological process, among which ATM/CHK2 and ATM RAD3-related/checkpoint kinase 1 (ATR/CHK1) are two main pathways responding to DSBs and SSBs, respectively [17]. Since we had already detected DSBs in host cells, we then examined whether ATM/CHK2 pathway was activated in the *T. gondii*-infected cells. ATM-S1981 and CHK2-T68 are the functionally activated form of ATM and CHK2, respectively [17]. The levels of ATM-S1981 and CHK2-T68 quantified by western blot were approximately 10-, 65-, 70-fold and 1-, 15-, 25-fold, respectively at 10, 20 and 30 hpi compared to the uninfected controls of 0 h (ATM-S1981 10 h: ANOVA: *F*_(3, 8)_ = 34.28, *P* = 0.0002; 20 h: ANOVA: *F*_(3, 8)_ = 34.28, *P* = 0.0003; 30 h: ANOVA: *F*_(3, 8)_ = 34.28, *P* = 0.0005; CHK2-T68 10 h: ANOVA: *F*_(3, 8)_ = 54.92, *P* = 0.9981; 20 h: ANOVA: *F*_(3, 8)_ = 54.92, *P* = 0.0003; 30 h: ANOVA: *F*_(3, 8)_ = 54.92, *P* = 0.0006) (Fig. 4a–c). This result indicated that the ATM/CHK2 pathway was activated during *T. gondii* infection.

## Discussion

DNA damage is classified as SSBs, DSBs, and chemical modification of bases. These different types of DNA damage can be detected by various markers. We used γH2AX to detect DNA DSBs in the *T. gondii* infected cells. Other types of DNA damage in the eukaryotic cells can be identified by corresponding suitable markers. For example, anti-8oxoG antibody can be used to detect chemical modification of DNA bases under oxidative stress [33, 34]. Here, DSBs were clearly demonstrated in the *T. gondii*-infected host cells. Evaluation of other types of DNA damage in these cells is beyond the scope of the present paper and waits to be further performed. Whether *T. gondii* cells themselves show DSBs is worthy of a brief discussion. We did not detect them in both western blot and IFA using the antibody to γH2AX. Nevertheless, a basal level of DSBs in proliferating *T. gondii* has been previously reported [35]. One plausible reason for this discrepancy is that the different monoclonal antibodies (mAb) to γH2AX are used in the two studies. Our mAb (20E3), was purchased from Cell Signaling Technology, whereas the mAb (JBW301) in the previous study was purchased from Merck Argentina. mAbs to the same protein may recognize different epitopes. Alternatively, this discrepancy may be due to the different binding capacity of the two mAbs.

It has been reported that *T. gondii* infection inhibits apoptosis of host cells [36]. According to our data, *T. gondii* infections do not cause apoptosis in its host cells which aligns well with these earlier reports even though they do not exactly confirm inhibition of apoptosis in the infected cells. In our experiment of blocking invasion of *T. gondii*, one shortcoming of using mouse serum to inhibit the invasion of host cells by *T. gondii* is that there might exist antibodies to the molecules that play important roles other than its invasion. Reagents that have been proven to block this parasite invasion are alternatives. This can be achieved by the reagent’s action on either the *T. gondii* tachyzoites or host cells. One example of the former is protease inhibitors such as BAY11-7082 [37]. The latter includes dynamin inhibitor such as dynasore, a small chemical compound that inhibits dynamin GTPase activity [38]. Due to the technical challenges these compounds were not used in the present study. Nevertheless, the mouse serum containing polyclonal antibodies used in the current study indeed block the parasites’ invasion of HeLa cells (see Additional file 1: Figure S1).

ROS are inhibited by activating antioxidant enzymes, such as catalase, superoxide dismutase and glutathione peroxidase, or by non-enzymatic antioxidants, such as glutathione and thioredoxin. ROS inhibitor NAC reacts with hypochlorous acid, hydroxyl radical and hydrogen peroxide but not superoxide anion [39]. Our data clearly demonstrated that ROS inhibition by NAC decreased γH2AX levels by a minimum of 70%, leaving a maximum of 30% γH2AX intact. This may be due to the action of superoxide anion, which can be addressed later in a follow-up study by its specific inhibitors such as eugenol, chalcone and derivative, 3’-isopropoxychalcone (H2O7D) [40, 41]. Our result showed that *T. gondii* infection causes a significant increase in the level of ROS in HeLa cells. Although consistent with some earlier reports [12, 31], these are clearly contradicted to at least one other study. Decreased levels of ROS were observed in *T. gondii*-infected ARPE-19 cells, a human RPE cell line, that had also been exposed to H_2_O_2_ treatment, and *T. gondii*-infected ARPE-19 cells at a MOI of 5:1 without H_2_O_2_ treatment showed ROS levels very similar to the uninfected controls [36]. These contradicting results might be due to different type of cells, i.e. HeLa *versus* ARPE-19, and different MOI of 10:1 and 5:1. Furthermore, DSBs was repaired in 1 h of ROS removal by NAC, the shortest time point used in the present study, as shown by a dramatical decrease of γH2AX levels. DSBs repair is very likely to occur and be observed in minutes if shorter time points had been included in our experiment.

Activation of the ATM/CHK2 pathway is involved in many biological processes such as DNA damage repair, cell cycle control and apoptosis etc. For example, the active ATM/CHK2 pathway leads to phosphorylation of P53, which is a well-known transcription factor. On one hand, phosphorylation of P53 at Ser25, Ser20 and Ser33 upregulates the expression of DNA binding protein 2, E3 ubiquitin-protein ligase MDM2 and CDC25a. The latter three are the key players in DNA damage repair and cell cycle control, which contribute to survival of wounded cells [42]. On the other hand, phosphorylation of P53 at Ser46 by ATM/CHK2 pathway activates pro-apoptotic genes, leading to apoptosis of wounded cells [43]. We propose that activation of the ATM/CHK2 pathway plays a pivotal role in the host cells to keep a calculated balance between survival and apoptosis during *T. gondii* infection, probably for the benefit of parasite survival.

Identification of parasite molecules causing host cell DNA damage and their molecular interactions with host cells are beyond the scope of the current study. However, it has been reported that *T. gondii* ROP18 kinase interacts with host proteins involving DNA repair and other functions [44]. Ocular and brain lesions caused by *T. gondii* infections are confirmed in human individuals with significant polymorphism of DNA repair genes [45]. Therefore, *T. gondii* virulence factors associated with DNA damage and host susceptibility to this damage are worth further investigation.

## Conclusions

Our studies clearly showed that *T. gondii* infection induced DNA double-strand breaks in host cells. This damage was irrelative to apoptosis and occurred only after *T. gondii* invasion. Reactive oxygen species were determined as the major player for DNA double-strand breaks in host cells. The host DNA damage response pathway ATM/CHK2 was activated during *T. gondii* infection, suggesting the DNA damage process may trigger other biological processes as well. More studies are needed to explore the molecular network of DNA double-strand breaks as well as other types of DNA damage in host cells in the future.

## Supplementary information

**Additional file 1: Figure S1.** Serum of *T. gondii*-infected BALB/c mice blocked *T. gondii* invasion to HeLa cells. EGFP-RHΔku80 tachyzoites were pre-incubated with either positive mouse serum collected at 6 days post-infection or negative serum harvested at 0 day prior to infection at a 1:10 dilution for 1 h. Afterwards, these parasites were added to HeLa cells at a multiplicity of infection (MOI) of 10:1 at 0 h when images were taken immediately (left panels). EGFP of the infected HeLa cells was again imaged at 24 hpi by fluorescence microscopy (right panels). Experiments were performed in triplicate. *Scale-bars*: 100 μm.

## Data Availability

Data supporting the conclusions of this article are included within the article and its additional file. Data and materials can be available upon reasonable request to the corresponding author.

## Abbreviations

MOI: Multiplicity of infection
ROS: Reactive oxygen species
NAC: N-acetylcysteine
IFA: Immunofluorescence assay
mAb: Monoclonal antibody
ATM/CHK2: Ataxia telangiectasia mutated/checkpoint kinase 2
SSBs: Single-strand breaks
DSBs: Double-strand breaks
FBS: Fetal bovine serum
DMEM: Dulbecco’s modified Eagleʼs medium
IMC1: Inner membrane complex 1
SAG1: Surface protein 1
TBS: Tris buffered saline
PBS: Phosphate-buffered saline
H2DCFDA: 2′,7′-dichlorodihydrofluorescein diacetate
