# Supplementary figures and images for: DNA double-strand breaks in the Toxoplasma gondii-infected cells by the action of reactive oxygen species
Source: Parasit Vectors. 2020 Sep 25;13:490. doi: 10.1186/s13071-020-04324-7 (PMC7523337; doi:10.1186/s13071-020-04324-7)

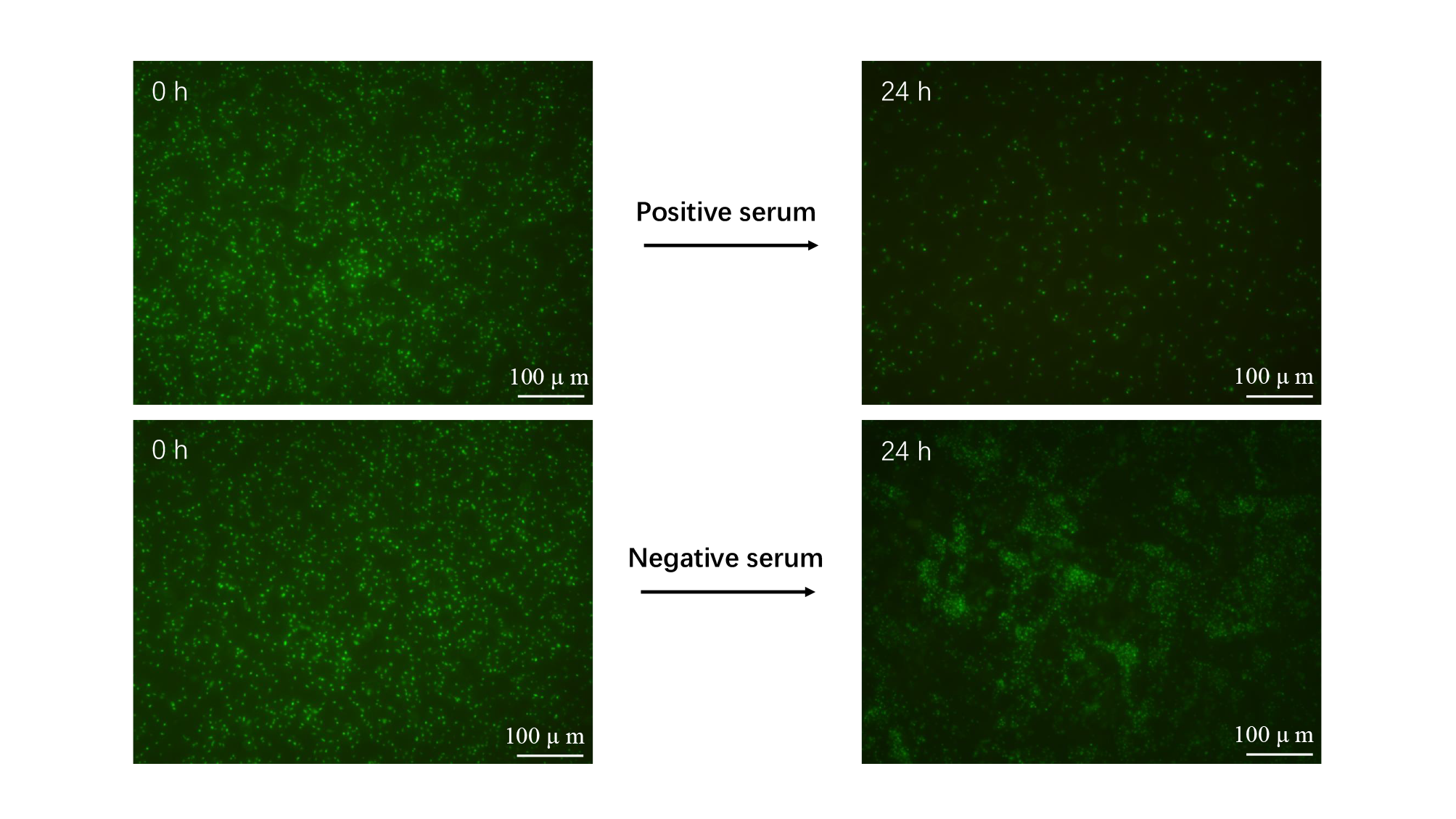

Supplement: Supplementary file 1 — Additional file 1: Figure S1. Serum of T. gondii-infected BALB/c mice blocked T. gondii invasion to HeLa cells. EGFP-RHΔku80 tachyzoites were pre-incubated with either positive mouse serum collected at 6 days post-infection or negative serum harvested at 0 day prior to infection at a 1:10 dilution for 1 h. Afterwards, these parasites were added to HeLa cells at a multiplicity of infection (MOI) of 10:1 at 0 h when images were taken immediately (left panels). EGFP of the infected HeLa cells was again imaged at 24 hpi by fluorescence microscopy (right panels). Experiments were performed in triplicate. Scale-bars: 100 μm. [file 13071_2020_4324_MOESM1_ESM.tif]
